# LncRNA SNHG15: A new budding star in human cancers

**DOI:** 10.1111/cpr.12716

**Published:** 2019-11-27

**Authors:** You Shuai, Zhonghua Ma, Jianwei Lu, Jifeng Feng

**Affiliations:** ^1^ Department of Medical Oncology Jiangsu Cancer Hospital Jiangsu Institute of Cancer Research The Affiliated Cancer Hospital of Nanjing Medical University Nanjing China; ^2^ Key Laboratory of Carcinogenesis and Translational Research (Ministry of Education) Division of Gastrointestinal Cancer Translational Research Laboratory Peking University Cancer Hospital and Institute Beijing China; ^3^ Department of Gastrointestinal Surgery Peking University Cancer Hospital and Institute Beijing China

**Keywords:** biomarker, cancer, LncRNA, molecular mechanism, SNHG15

## Abstract

**Objectives:**

Long non‐coding RNAs (lncRNAs) represent an important group of non‐coding RNAs (ncRNAs) with more than 200 nucleotides in length that are transcribed from the so‐called genomic “dark matter.” Mounting evidence has shown that lncRNAs have manifested a paramount function in the pathophysiology of human diseases, especially in the pathogenesis and progression of cancers. Despite the exponential growth in lncRNA publications, our understanding of regulatory mechanism of lncRNAs is still limited, and a lot of controversies remain in the current lncRNA knowledge.The purpose of this article is to explore the clinical significance and molecular mechanism of SNHG15 in tumors.

**Materials & Methods:**

We have systematically searched the Pubmed, Web of Science, Embase and Cochrane databases. We provide an overview of current evidence concerning the functional role, mechanistic models and clinical utilities of SNHG15 in human cancers in this review.

**Results:**

Small nucleolar RNA host gene 15 (SNHG15), a novel lncRNA, is identified as a key regulator in tumorigenesis and progression of various human cancers, including colorectal cancer (CRC), gastric cancer (GC), pancreatic cancer (PC) and hepatocellular carcinoma (HCC). Dysregulation of SNHG15 has been revealed to be dramatically correlated with advanced clinicopathological factors and predicts poor prognosis, suggesting its potential clinical value as a promising biomarker and therapeutic target for cancer patients.

**Conclusions:**

LncRNA SNHG15 may serve as a prospective and novel biomarker for molecular diagnosis and therapeutics in patients with cancer.

## 1 INTRODUCTION

A significant breakthrough of the Encyclopedia of DNA Elements (ENCODE) project is that only ~3% of the genome contains protein‐coding genes, and more than 90% of the human genome is actively transcribed.^1^, ^2^ Non‐coding RNAs (ncRNAs) constitute an overwhelmingly high percentage (≥ 80%) of human transcripts.^3^ Long non‐coding RNAs (lncRNAs) are a class of ncRNAs longer than 200 nucleotides in length that are transcribed by RNA polymerase II and lack open reading frame.^4^ This new class of regulators were initially considered to be transcriptional noise with no specific biological functions.^5^ Further research confirmed that sets of lncRNAs, with a high degree of evolutionary conservation, are identified to be biologically functional.^6^

Recent evidence shows that lncRNAs are identified as critical regulators in various biological processes, such as cell growth, cell cycle, cell apoptosis, cell differentiation, cell invasion and metastasis.^7^, ^8^, ^9^, ^10^ These lncRNAs could serve as transcriptional regulator, splicing mediator, post‐transcriptional processor, competing endogenous RNAs (ceRNAs) for miRNAs and chromatin remodeler in tumorigenesis and progression.^8^, ^11^, ^12^ Based on the targeting mechanism, lncRNAs could be classified as signal, scaffold, decoy and enhancer.^3^ Interestingly, lncRNAs provide a novel way of regulating the gene expression at transcriptional, post‐transcriptional, and epigenetic levels.^10^, ^13^, ^14^, ^15^, ^16^ Additionally, lncRNAs can regulate a variety of cancer‐related signalling networks though interacting with protein, such as wnt/β‐catenin signalling pathway, epithelial‐mesenchymal transition (EMT) signalling pathway, NF‐kB signalling pathway and Hippo signalling pathway.^17^, ^18^, ^19^, ^20^ For instance, linc00673 augmented the binding between DEAD box RNA helicase DDX3 and casein kinase 1ε (CK1ε), thus activating wnt/β‐catenin signalling and causing aggressiveness of lung adenocarcinoma (LAD).^21^

Accumulating studies demonstrate that dysregulated lncRNA expression could exert oncogenic or tumour‐suppressive roles in cancer progression.^3^, ^22^ For example, metastasis‐associated lung adenocarcinoma transcript 1 (MALAT1), as a well‐known lncRNA, has been shown to be either upregulated or downregulated in human cancers.^3^, ^22^ Previous in‐vitro assay and xenograft studies revealed contradictory effects of MALAT1 on cancer phenotype.^23^, ^24^, ^25^, ^26^, ^27^, ^28^ Similarly, lncRNA SNHG15 was previously described by many papers as a cancer‐promoting and metastasis‐promoting lncRNA, while other report demonstrated a tumour‐suppressing function of SNHG15.^29^, ^30^, ^31^, ^32^, ^33^ Its dysregulation was closely correlated with carcinogenesis, affecting the prognosis of cancer patients.^34^, ^35^, ^36^ Although these inconsistent findings could be of the limitation in sample size or the difference in tumour origin but also highlight the necessity of more research in SNHG15 in various types of cancer. Recently, it is notable that the aberrant gene expression in circulating tumour cells (CTCs) can influence the prognosis of cancer patients, such as mesenchymal markers.^37^ Cai et al identified the status of cyclooxygenase‐2 (COX‐2) expression in CTCs and revealed its correlation with clinical and pathological factors of CRC patients.^38^ This interesting observation may also remind us to explore the molecular basis of other critical genes in CTCs, such as lncRNAs, thus establishing theoretical foundation for future translational research.

In this review, we aim to summarize the latest knowledge about the characteristics of SNHG15 in the biological effects and molecular mechanism of human cancers and further debate the prognostic and therapeutic values of SNHG15 in human cancers.

## 2 IDENTIFICATION AND CHARACTERIZATION OF lncRNA SNHG15

SNHG15, a strongly conserved lncRNA which was initially reported in 2012, is located at 7p13 with a length of 860 bp.^39^ Studies raised the possibility that the stability of ncRNAs could reflect their potential function, based on the existed findings between the half‐life of each mRNA and its physiological function.^39^ Originally, a novel genome‐wide method, 5'‐bromo‐uridine immunoprecipitation chase‐deep sequencing analysis (BRIC‐seq), was used to determine the half‐lives of ncRNAs and mRNAs in HeLa Tet‐off (TO) cells.^39^ Through analysing the relationship between the half‐life of RNA and functional categories, RNAs with a short half‐life (*t* (1/2) < 4 hours) contained known regulatory ncRNAs and regulatory mRNAs.^39^ LncRNA SNHG15 was screened out as a short‐lived non‐coding transcripts (SLiTs) with a short half‐life (*t* (1/2) < 4 hours), and likely to be involved in cell proliferation.^39^ Additionally, the prediction of SNHG15 structure based on minimum free energy (MFE) and partition function can be obtained from RNA‐fold website(http://rna.tbi.univie.ac.at//cgi-bin/RNAWebSuite/RNAfold.cgi?PAGE=3%26ID=TryBo7KkMy).

Further research indicated that SNHG15, as a critical member of short‐lived lncRNAs, participated in the molecular mechanisms associated with responses to cellular stresses.^39^ The expression level of SNHG15 was elevated due to prolonged decay rates in response to chemical stressors and interruption of RNA degradation pathways.^39^ It has been proposed that SNHG15 has the potential to be surrogate indicators of cellular stress responses.^39^ Of note, SNHG15 was dysregulated in various tumour tissues and cell lines, such as CRC, GC, pancreatic cancer (PC) and thyroid cancer (TC).^19^, ^32^, ^33^, ^40^, ^41^ Several aspects of tumorigenicity, such as cell proliferation, apoptosis, migration and metastasis, have been evaluated in relation to SNHG15 expression in human cancers.^30^, ^42^, ^43^, ^44^ In addition, aberrant SNHG15 expression displayed close correlation with tumour size, tumour node metastasis (TNM) stage, lymph node metastasis and prognosis of cancer patients.^29^, ^35^, ^36^, ^42^ The expression pattern, functional role and regulatory mechanism of SNHG15 are presented in Table 1.

**Table 1:** LncRNA SNHG15 in human cancers

Cancer types	Expression	Role	Clinical correlation	Functional role	Regulatory molecules and pathways
Colorectal cancer	Upregulated	Oncogenic	Tumour size, TNM stage, lymph node metastasis, liver metastasis, CEA, OS	Proliferation, apoptosis, migration, invasion, 5‐Fu resistance, colorectal liver metastasis	SNHG15/miR‐141/SIRT1, SNHG15/miR‐338‐3p/FOS/RAB14, MYC/SNHG15/AIF/ROS, SNHG15/Slug/Upp,Wnt/β‐catenin signalling pathway
Gastric cancer	Upregulated	Oncogenic	Invasion depth, TNM stage, lymph node metastasis, OS, DFS	Proliferation, migration, invasion, apoptosis	SNHG15/MMP2/MMP9
Pancreatic cancer	Upregulated	Oncogenic	Tumour size, TNM stage, lymph node metastasis, differentiation degree	Proliferation, cell cycle, apoptosis	SNHG15/EZH2/P15/KLF2
Hepatocellular carcinoma	Upregulated	Oncogenic	Histological grade, TNM stage, vein invasion, OS	Proliferation, cell cycle, migration, invasion	SNHG15/miR‐141‐3p/ZEB2/E2F3
Lung cancer	Upregulated	Oncogenic	Tumour size, lymph node status, TNM stage, OS, DFS	Proliferation, apoptosis, migration, invasion, metastasis, EMT	SNHG15/MMP2/MMP9, SNHG15/miR‐211‐3p, SNHG15/miR‐486/CDK14
Prostate cancer	Upregulated	Oncogenic	/	Migration, invasion, EMT	SNHG15/miR‐338‐3p/FKBP1A
Osteosarcoma	Upregulated	Oncogenic	/	Proliferation, migration, invasion, autophagy	SNHG15/miR‐141, SNHG15/Atg5/LC3‐I/ LC3‐II/p62
Glioma	Upregulated	Oncogenic	OS	Proliferation, migration, tube formation, angiogenesis, temozolomide resistance	SNHG15/miR‐153/VEGFA/Cdc42, SNHG15/CDK6/miR‐627
Breast cancer	Upregulated	Oncogenic	Tumour size, TNM stage, lymph node metastasis	Proliferation, apoptosis, migration, invasion, EMT	SNHG15/miR‐211‐3p/ZNF217
Renal cell carcinoma	Upregulated	Oncogenic	Histological differentiation, T stage, survival	Proliferation, migration, invasion, cell cycle, apoptosis, EMT	SNHG15/ N‐cadherin/Vimentin/E‐cadherin, NF‐κB signalling pathway
Ovarian cancer	Upregulated	Oncogenic	Cancer type, ascites, FIGO stage, OS, DFS	Proliferation, migration, invasion, chemoresistance	/
Thyroid cancer	Downregulated	Anti‐cancer	Age, pathology classification, clinical stage, tumour size, distant metastasis, OS, DFS	Proliferation, migration, invasion, EMT	SNHG15/miR‐510‐5p
Thyroid cancer	Upregulated	Oncogenic	Gender, tumour size, TNM stage, lymph node metastasis, OS	Proliferation, apoptosis, migration, EMT	SNHG15/miR‐200a‐3p/YAP1/Hippo signalling pathway

## 3 EXPRESSION PATTERN, FUNCTIONS AND CLINICAL POTENTIALS OF SNHG15 IN HUMAN CANCERS

### 3.1 Colorectal cancer

CRC is the third most common cancer, with approximately 1.3 million new cancer cases and 690,000 mortalities worldwide each year.^45^ Despite tremendous progress in the treatment of CRC in recent decades, the prognosis remains unsatisfactory, especially in advanced‐stage tumours with distant metastasis.^45^, ^46^ Current evidences show that approximately 25% of CRC patients present with synchronous liver metastases at diagnosis.^47^ The survival and prognosis of these patients with liver metastasis is extremely poor, with a 5‐year survival rate less than 10%.^47^, ^48^ Thus, it is extremely necessary to gain a better understanding of carcinogenesis and identify novel and sensitive biomarkers for diagnosis and treatments in CRC patients.

LncRNA SNHG15 has been found in several studies to be dramatically upregulated in CRC tissues and cells.^19^, ^49^, ^50^, ^51^ CRC patients with larger tumour size, advanced TNM stage and lymph node metastasis show higher SNHG15 expression.^19^, ^49^, ^50^ Furthermore, higher SNHG15 expression is correlated with a worse survival.^42^, ^50^, ^51^ Both univariate and multivariate analysis confirmed that SNHG15 expression was significantly associated with overall survival (OS) in CRC patients, suggesting that SNHG15 could be used as an independent prognostic factor and potential biomarker for CRC patients.^42^, ^50^, ^51^ Functional assays further demonstrated that knockdown of SNHG15 could inhibit CRC cell proliferation, activate cell apoptosis and suppress cell migration and invasion both in vitro and in vivo.^19^, ^49^, ^50^, ^51^ Recently, SNHG15 has been shown to present a downside tendency on regulating miR‐141, which downregulates sirtuin 1 (SIRT1) in CRC cells. The promotion effects of tumour growth and metastasis driven by SNHG15 overexpression can be significantly reversed by miR‐141 inhibitor.^19^ Besides, wnt/β‐catenin signalling pathway was found to be involved in SNHG15‐mediated carcinogenesis and could act as a downriver regulator in CRC.^19^ These findings suggest that SNHG15/miR‐141/SIRT1 axis exerts oncogenic functions in CRC.^19^ Similarly, Min et al found that SNHG15 mediated CRC proliferation through SNHG15/miR‐338‐3p/FOS/RAB14 axis.^19^

Interestingly, Saeinasab et al demonstrated that SNHG15 is more highly expressed in CRC tissues with high levels of MYC expression.^49^ Mechanistically, MYC protein binds to two E‐box motifs on SNHG15 sequence, illuminating that the transcription of SNHG15 is directly activated by the oncogene MYC.^49^ In‐vitro assays found that decreased SNHG15 expression could inhibit tumorigenic capacity of CRC cells and increased SNHG15 expression could display opposite effects.^49^, ^50^ Besides, ROS levels, which are directly regulated by AIF protein, were obviously decreased in CRC cells with SNHG15 knockdown, and SNHG15 could bind with AIF protein in CRC cells, suggesting that the activity of AIF was partially mediated by SNHG15.^49^ Moreover, Jiang et al reported that SNHG15 can mediate the stability of Slug, which is a critical transcription factor involved in tumorigenesis, EMT, embryonic development and stem cell reprogramming.^51^, ^52^, ^53^, ^54^The stability of Slug determines its biological functions and can be controlled by various regulatory mechanism, including the interaction with lncRNAs.^55^, ^56^, ^57^ Jiang et al uncovered that SNHG15 can interact with Slug and inhibit its degradation via the ubiquitin‐proteasome pathway (UPP).^51^ As a stabilizer of Slug, SNHG15 can also participate in the process of EMT.^51^ As for the impacts of SNHG15 on drug resistance, it was shown that SNHG15 overexpression could increase the resistance to 5‐Fu, while its downregulation could activate the sensitiveness to 5‐FU in CRC cells.^49^

There is increasing evidence that lncRNAs play significant roles in the regulation of colorectal liver metastasis (CLM).^58^, ^59^, ^60^ Another study reported by Huang et al have been illuminated the potential role of SNHG15 in CLM.^42^ Huang et al used the transcriptome sequencing (RNA‐seq) assay to determine significantly altered lncRNAs through analysing data among primary CRC lesions, synchronous liver metastatic lesions and adjacent normal mucosa.^42^ Based on the results of RNA‐seq and quantitative reverse‐transcription polymerase chain reaction (qRT‐PCR) assays, SNHG15 was found to be overexpressed in liver metastases lesions than that of the paired normal mucosa and primary CRC lesions. Besides, Huang et al further performed qRT‐PCR assays in the independent and extended validation cohort of 91 pairs of normal adjacent mucosa and primary CRC tissue (50 without liver metastasis and 41 with liver metastasis) to test the SNHG15 expression level.^42^ Intriguingly, the expression level of SNHG15 exhibited significant increase in primary CRC lesions with liver metastasis compared with that of primary CRC lesions without liver metastasis.^42^ Moreover, SNHG15 overexpression was remarkably associated with liver metastasis, carcinoembryonic antigen (CEA), TNM stage and lymph node metastasis.^42^ These findings suggested that lncRNA SNHG15 may be involved in the liver metastasis processes of CRC. All the above results show that SNHG15 can promote CRC development and progression through various molecular mechanisms.

### 3.2 Gastric cancer

GC is the third leading cause of cancer‐related death around the world.^61^ It is extremely a huge burden worldwide with high morbidity and high mortality, especially in East Asia.^61^, ^62^ The overall 5‐year relative survival rate for advanced GC patients is still lower than 5%.^63^ It is also known that most patients with GC have reached their advanced stage at initial diagnosis because of the lack of novel molecular biomarkers for diagnosis.^63^, ^64^ Thus, it has been a central issue to clarify the regulatory mechanism critical for the GC carcinogenesis and tumorigenesis. More importance should be attached to identify effective biomarkers for GC diagnosis and treatment.

SNHG15 was firstly recognized as a novel prognostic factor in GC patients.^63^, ^64^ Chen et al tested the expression level of SNHG15 in 106 pairs GC tissue and matched adjacent non‐tumour tissues using qRT‐PCR assay.^40^ The results showed that SNHG15 expression levels were higher in GC tissues than in the corresponding non‐cancerous tissues, and there was a close correlation between SNHG15 expression and clinical‐pathological factors in GC patients. High SNHG15 expression was closely associated with invasion depth, TNM stage and lymph node metastasis in patients with GC.^40^ Importantly, the expression level of SNHG15 was also in close correlation with OS and disease‐free survival (DFS). Kaplan‐Meier analysis demonstrated that elevated SNHG15 expression contributed to poorer OS and DFS of GC patients.^40^ And multivariate survival analysis validated that SNHG15 could be an independent prognostic marker of OS and DFS in GC patients.^40^

Besides, SNHG15 was overexpressed in GC cells, compared with GES‐1 cell.^40^ Upregulation of SNHG15 contributes to tumour‐promoting activities, while its downregulation plays tumour‐suppressing functions in GC. Chen et al revealed that knockdown of SNHG15 suppressed cell proliferation and invasion and induced a strong apoptotic response in GC cells.^40^ Additionally, SNHG15 amplification could promote GC cell proliferation and invasion and largely increase the expression level of matrix metallopeptidase 2 (MMP2) and matrix metallopeptidase 9 (MMP9) at protein levels.^40^ These results showed that elevated expression of SNHG15 could facilitate GC development and progression partly through modulating MMP2 and MMP9.^40^ However, the molecular mechanism between SNHG15 and MMP2/MMP9 is not clear. Further investigations are required to determine the effects of SNHG15 on tumour growth and metastasis in vivo and to illuminate the exact regulatory mechanism of SNHG15 in GC progression.

### 3.3 Pancreatic cancer

PC is the fourth most common cause of cancer mortality worldwide, leading to approximately 227 000 deaths annually.^65^, ^66^ The 5‐year relative survival of PC remained at approximately 8% for 2005‐2011.^65^ PC is proposed to be one of the top two cancers in terms of fatalities in the next decade.^67^ Surgical resection remains the exclusive potential curative treatment.^68^ However, approximately half of patients present with metastasis at the time of diagnosis, missing the opportunity for treatment.^68^, ^69^ A growing body of literature has demonstrated that metastasis and limited effective biomarker for diagnosis and treatment are the main obstacles for PC medical therapy.^69^, ^70^, ^71^ Thus, it is an absolute necessity to identify potential biomarker and therapeutic target in PC.

Ma et al have highlighted the oncogenic effects of SNHG15 in PC initiation and progression.^41^ The elevation of SNHG15 was indicated to be closely correlated with tumour size, TNM stage and lymph node metastasis in PC patients, revealing its potential as a promising biomarker.^41^ Another study by Gao et al showed that overexpression of SNHG15 was positively associated with differentiation degree, TNM stage and lymph node metastasis in patients with pancreatic ductal adenocarcinoma (PDAC).^29^ ROC curve analysis showed that SNHG15 level could distinguish PDAC tissues from normal pancreatic tissues (AUC = 0.785), presenting the diagnostic value of SNHG15 in PC.^29^ The optimal cut‐off value for SNHG15 in PDAC tissues was 5.38 with a sensitivity of 71.7% and a specificity of 92.6%. However, no significant correlation was found between tumour size and SNHG15 expression, which is contrary to the results published by Ma et al The limited tissue samples and different sources of tissue samples can partly account for this inconsistent finding. Additionally, Gao et al further revealed the high SNHG15 expression level in the sera from PDAC patients, compared with healthy controls.^29^ The serum SNHG15 expression level could act as a potential biomarker for screening PDAC patients from healthy controls (AUC = 0.727). The optimal cut‐off value for SNHG15 in sera of PDAC patients was 6.82 with a sensitivity of 68.3% and a specificity of 89.6%.^29^ Kaplan‐Meier analysis revealed that the 5‐year OS rate for high SNHG15 group was lower than those in the low SNHG15 group.^29^ Based on the univariate analysis and multivariate Cox proportional models, SNHG15 was screened out as an independent prognostic factor for patients with PDAC.^29^

As a dysregulated lncRNA in PC, knockdown of SNHG15 could impair PC cell proliferation, cause G1/G0 phase arrest and activate apoptosis.^41^ Besides, overexpression of SNHG15 was observed to boost the proliferative capacity of PC cells through regulating cell cycle and apoptosis correlated proteins, presenting as the reduction of P15 and KLF2.^41^ The SNHG15 impact can be partly reversed by overexpression of P15 or KLF2.^41^ Consistently, SNHG15 exhibited a negative correlation with P15 or KLF2 in PC tissues. Upon in‐vivo assay, SNHG15 knockdown could exhibit suppressive effects on tumour growth in PC.^41^ Mechanistically, a SNHG15/EZH2/P15/KLF2 axis was identified in PC, shedding new light on lncRNA‐based diagnosis and therapeutic in PC.^41^

### 3.4 Hepatocellular carcinoma

Liver cancer is the sixth most common cancer and the fourth leading cause of cancer‐related death worldwide in 2018.^61^ It is predicted that 841 000 new cases and 782 000 deaths occur annually.^61^ HCC accounts for 75%‐80% of all liver cancer cases, and half of the cases are discovered in China.^61^, ^72^ Based on the huge population, HCC poses a big burden for human health worldwide, especially for China.^72^ Approximately 70% of patients will relapse and 30% patients will suffer tumour‐related death within 5 years after liver resection.^73^ Therefore, the mechanistic investigation of hepatocarcinogenesis and identification of more efficient molecular markers and therapeutic target are urgently needed for HCC patients.

Zhang et al reported that SNHG15 was significantly elevated in a cohort of 152 pairs HCC tissues and adjacent normal tissue, suggesting SNHG15 may be a critical regulator in HCC tumorigenesis.^34^ High SNHG15 expression was found to be associated with clinicopathological parameters, such as histological grade, TNM stage, and vein invasion using chi‐square analysis. To determine the correlation between SNHG15 level and prognosis of HCC patients, Kaplan‐Meier analysis, univariate analysis, as well as multivariate Cox regression analysis were used. It was shown that SNHG15 elevation was positively correlated with poor overall survival of HCC patients and SNHG15 can serve as an independent prognostic indicator for HCC patients.^34^ This study conducted by Zhang et al highlighted the potential of SNHG15 as an oncogene in HCC pathogenesis.

Current study reported by Ye et al confirmed that SNHG15 can play promoting role in regulating HCC proliferation, migration and invasion.^34^ Moreover, knockdown of SNHG15 can result in a significant increase in G1/G0 phase and an obvious decrease in S phase. Mechanically, SNHG15 can mediate the expression level of ZEB2 and E2F3 through sponging miR‐141‐3p in HCC cells, thus promoting HCC progression.^74^ This study may offer new insights regarding HCC pathology and provide potential strategy for lncRNA‐directed treatment. However, the in‐vivo influence and other underlying mechanism of SNHG15 still determine to be clarified in the future research.

### 3.5 Lung cancer

Lung cancer is the leading cause of cancer‐related deaths worldwide.^61^ Non‐small cell lung cancer (NSCLC) accounts for approximately 85% of lung malignancies and includes lung adenocarcinoma (LUAD), squamous cell carcinoma and large cell lung cancer.^61^, ^75^, ^76^ Although there are various ways for diagnosis and treatments, the rate of 5‐year OS of advanced lung cancer patients is less than 15%.^77^ Therefore, it is essential to find valuable tumour makers for early diagnosis and therapy.

Recently, lncRNA SNHG15 emerges as an important regulator in lung cancer. Dong et al revealed that SNHG15 was highly elevated in NSCLC tissues compared with controls.^36^, ^78^ Analysis of correlation between SNHG15 expression and clinicopathological data showed that high expression level of SNHG15 was positively related to the tumour size, lymph node status and TNM stage in NSCLC patients.^36^, ^78^ Kaplan‐Meier survival analysis of OS and DFS revealed that NSCLC patients with higher SNHG15 expression had a relatively poor prognosis compared with the low SNHG15 group.^36^, ^78^ The information regarding SNHG15 expression may be useful to predict the survival of NSCLC patients. In‐vitro assays revealed that decreased SNHG15 expression could obviously impair proliferative capacity of NSCLC cells, cause G0/ G1 phase arrest and increase the ratio of apoptotic NSCLC cells.^36^, ^78^ Moreover, downregulation of SNHG15 substantially inhibited the invasive and metastatic ability of NSCLC cells.^36^, ^78^ The pro‐metastatic effects of SNHG15 were induced by the regulation of the expression of a number of genes involved in cell metastasis and EMT progress. Depletion of SNHG15 was involved in the downregulation of MMP2 and MMP9 expression, while the underlying mechanism was still unclear.^36^ Another study discovered that SNHG15 induced lung cancer proliferation through regulating miR‐211‐3p, which was predicted to interact with SNHG15. Further research confirmed that miR‐211‐3p can bind to SNHG15 and its downregulation can partly rescued the proliferation promotion driven by SNHG15 overexpression in A549 and H1799 cells.^78^ Moreover, SNHG15 could upregulate CDK14 expression via sponging miR‐486, thus contributing to NSCLC tumorigenesis.^44^ These findings elucidated that SNHG15 can activate the malignant phenotypes of NSCLC cells through a mechanism involving miRNAs. However, more efforts should be devoted to clarifying other regulatory mechanisms and clinical implication of SNHG15 in lung cancer in the future.

### 3.6 Thyroid cancer

TC continues to be the most common endocrine malignant tumour and has emerged as a major health issue.^79^ It is estimated that more than 60 000 people of TC occur annually in the United States.^79^, ^80^ TC is the sixth most common malignant tumour in the female population of China, where the incidence of TC is about 6.6 per 100 000 people.^81^ The major subtypes of TC include papillary thyroid cancer (PTC), follicular thyroid cancer (FTC), poorly differentiated thyroid cancer (PDTC) and anaplastic thyroid cancer (ATC) originate from follicular cell‐derived thyroid cells. PTC accounts for more than 85% of TC patients, and approximately 10%‐15% of patients with PTC exhibit relapse and metastasis after therapy, leading to poor outcome.^83^ Among these subtypes, ATC is the most aggressive and deadly thyroid cancer with only 3‐5 months overall survival after initial diagnosis.^83^ The studies of molecular mechanism correlated with TC greatly facilitate the understanding of TC cancer pathogenesis.^82^, ^84^, ^85^ Thus, it is of great significance to identify potential biomarkers and therapeutic targets involved in TC tumorigenesis.

Expression profile data of various cancers from The Cancer Genome Atlas (TCGA) data set showed that only TC specimens displayed lower levels of SNHG15 expression.^32^ Liu et al validated the expression status of SNHG15 in a cohort of 40 paired TC tissues and adjacent non‐tumour samples using qRT‐PCR assay.^32^ Compared with non‐cancerous TC samples, the expression level of SNHG15 was lower in TC tissue samples.^32^ Low expression level of SNHG15 was identified to closely associate with age, pathology classification, clinical stage, tumour size and distant metastasis in TC patients.^32^ However, current evidences mainly focus the overexpression of SNHG15 in most cancers. Tumour heterogeneity and limited TC samples can partly explain this inconsistent finding. Then, Kaplan‐Meier analysis and log‐rank test were conducted to estimate the prognostic efficiency of the candidate lncRNA SNHG15 in thyroid cancer patient's cohort from TCGA database. Intriguingly, it was demonstrated that the high level of SNHG15 expression was positively correlated with higher DFS rate in TC patients, while no significant association was found between OS and SNHG15 level in TC patients from TCGA data set.^32^ LncRNA SNHG15 may have a potential to be a novel biomarker for diagnosis and prognosis of TC.

Similarly, SNHG15 was significantly decreased in TC cell lines compared with normal thyroid cell and able to mediate tumour initiation, proliferation and metastasis in TC.^32^ The findings elucidated that increased SNHG15 expression dramatically repressed cell proliferation, migration and invasion in TC, revealing the tumour‐suppressive role of SNHG15 in TC.^32^ Another study identified that SNHG15 could act as a key target of miR‐510‐5p, which was proposed to be an oncogenic regulator in TC tumorigenesis.^86^ And a negative correlation between SNHG15 and miR‐510‐5p expression was revealed in TC tissues.^86^ Mechanistically, miR‐510‐5p directly interacted with SNHG15 and obviously repressed SNHG15 expression, thus contributing to TC cell proliferation, migration and invasion.^86^

Interestingly, a recent study uncovered by Wu et al also focused on the biological role of SNHG15 in PTC.^33^ Wu and his colleagues tested the expression levels of SNHG15 in 92 paired PTC tissues and corresponding normal tissues.^33^ Inconsistently, an obvious upregulation of SNHG15 was found in PTC tissues compared with control group. SNHG15 elevation was positively correlated with gender, larger tumour size, advanced TNM stage and positive lymph node metastasis.^33^ Meanwhile, high SNHG15 expression level was negatively correlated with the OS rate of PTC patients, suggesting its prognostic value for PTC patients.^33^ Knockdown of SNHG15 can dramatically activate apoptosis and obviously suppress cell proliferation migration and EMT progress in PTC.^33^ In addition, YAP1 is known as a core regulator of Hippo signalling pathway, which can be inactivated by SNHG15.^33^ Mechanistic investigation showed that SNHG15 can significantly increase oncogenic YAP1 expression through sponging miR‐200a‐3p in PTC cells, thus leading to PTC development and progression.^33^ The effects of SNHG15 on TC progression reported by two research teams are completely opposite to each other. Moreover, the role of SNHG15 conducted by Liu et al was contrary to the mechanistic model of SNHG15 observed in other malignancies.^33^, ^86^ The conflict of SNHG15 existed in TC and other types of cancer was partly due to limited numbers of tumour samples, different sample sources and tumour heterogeneity. Meanwhile, more animal experiments are needed to verify the role of SNHG15 in cancer growth and metastasis in vivo.

### 3.7 Prostate cancer

Prostate cancer (PC) is the most common malignancy in male, with more than 29 000 men killed by PC in 2018 in the united states.^65^ Owing to lack of specific and sensitive methods for early prostate cancer screening, most patients have been in the terminal stage at first diagnosis, with a 5‐year survival rate of only 29% in PC patients.^65^, ^87^ Serum prostate specific antigen (PSA), as an essential serum marker, has been widely utilized in the early detection and subsequent treatment.^88^ However, PSA was not specifically correlated with malignancy and can alter frequently and inconsistently depending on the circumstance.^88^, ^89^ Thus, uncovering new diagnostic biomarker and therapeutic target for PC patients is in an urgent need.

Zhang et al examined the expression levels of SNHG15 in PC cells and found that SNHG15 was significantly increased in PC cell lines.^88^, ^89^ SNHG15 silencing reduced prostate cancer cell growth in vitro and in vivo.^30^ Besides, knockdown of SNHG15 inhibited the migratory and invasive abilities of PC cells.^30^ Interestingly, SNHG15 reversed the progress of EMT presenting as reduction of E‐cadherin and enrichment of N‐cadherin, highlighting the involvement of SNHG15 in EMT regulation. Mechanistic studies showed the cytoplasmic localization of SNHG15 in Prostate cancer cells, suggesting the regulatory mechanism of SNHG15 at the post‐transcription level.^30^ SNHG15 could act as a molecular sponge to modulate miR‐338‐3p, which can potentially target FKBP prolyl isomerase 1A (FKBP1A) in tumorigenesis.^30^ Aside from that, miR‐338‐3p decrease and FKBP1A increase can obviously reversed the inhibition of cell migration and invasion mediated by SNHG15 knockdown.^30^ It suggested that targeting the SNHG15/miR‐338‐3p/FKBP1A axis may represent a novel therapeutic application in PC.^30^ However, the expression pattern of SNHG15 in prostate cancer is not clear, and the correlation between SNHG15 expression and clinical factors, as well as the prognostic value of SNHG15, should be further investigated in the future.

### 3.8 Osteosarcoma

Osteosarcoma (OS) functions as one of the most common primary bone malignancies among adolescent cohort.^30^, ^90^ Fast growth and early metastasis are the main reasons accounting for the dismal prognosis of osteosarcoma.^91^ Approximately 20% patients have pulmonary metastasis in the first visit.^92^ Thus, it is urgently needed to discover novel biomarker and therapeutic target for OS patient and better understand the mechanism of OS pathogenesis.

Liu et al demonstrated that SNHG15 was remarkably increased in OS tissues.^31^ In contrast to the expression pattern of SNHG15 in OS, miR‐141 was obviously downregulated in OS tissues.^31^ In addition, an inverse correlation was revealed between SNHG15 and miR‐141 expression in OS tissues.^31^ Similarly, SNHG15 was shown to be overexpressed in OS cells, compared with osteoblastic cell HFOB1.^31^ Then, gain‐of and loss‐of assays were conducted to examine the effects of aberrant expression of SNHG15 on OS cell viability, migration and invasion. Knockdown of SNHG15 could effectively inhibit OS cell proliferation, whereas its elevation obviously increase the proliferative capacity of OS cells.^31^ Silencing of SNHG15 strongly inhibited the migratory and invasive abilities of OS cells, compared with that in the control cells.^31^ And the migratory and invasive ability was activated by overexpression of SNHG15.^31^ In addition, SNHG15 was further determined to be associated with autophagy. Currently, mounting studies have identified reliable indicators of autophagy, such as autophagy related proteins (Atg5), cytosolic form of key protein LC3 in autophagosome formation (LC3‐I), active membrane‐bound form of LC3 (LC3‐II), and p62.^93^, ^94^, ^95^, ^96^ Liu et al uncovered that knockdown of SNHG15 can obviously elevate p62 expression and significantly decrease the expression level of Atg5, LC3‐II and LC3‐II/ LC3‐I ratio, illuminating the suppressive effects of SNHG15 knockdown on OS autophagy.^31^ Meanwhile, overexpression of SNHG15 displayed opposite effects on the expression level of Atg5, LC3‐II, LC3‐II/ LC3‐I ratio and p62, indicating that ectopic SNHG15 can activate autophagy in OS cells.^31^

Further research demonstrated that SNHG15 can interact with miR‐141 and dramatically downregulate the expression of miR‐141 in OS cells.^31^ The above results revealed that SNHG15 play a promoting role in OS cell proliferation, migration, invasion and autophagy, which was contrary to the influence of miR‐141 enrichment.^31^ It suggested that overexpressed miR‐141 can effectively reverse the promotion of OS tumorigenesis and autophagy induced by SNHG15 overexpression.^31^ Liu and his colleague proposed a mechanistic model that SNHG15 promotes OS tumorigenesis and autophagy partly through negatively regulating miR‐141.^31^ The SNHG15/miR‐141 axis may provide a potential marker and target for OS patients.^31^ However, the expression level of SNHG15 was only tested in 35 pairs OS tissue and matched non‐cancerous tissues. More OS samples should be involved to determine the expression pattern of SNHG15. Importantly, the correlation between SNHG15 level and clinicopathologic feature or survival of OS patients was still unclear. In the future, researchers should attach more importance on investigating the clinical significance, prognostic value, as well as mechanistic model of SNHG15 in OS development and progression.

### 3.9 Glioma

Glioma is one of the most prevalent types of primary intracranial carcinoma.^97^ It can be classified as grade II and grade III astrocytic tumours, oligodendroglioma, the grade IV glioblastoma (GBM) and diffuse glioma of childhood.^97^ Glioma is characterized by rapid cell proliferation and angiogenesis.^98^, ^99^, ^100^ Despite progress in diagnosis and therapy, the rate of recurrence and mortality 101is still high.^97^ Thus, extensive attention should be paid to finding prospective biomarkers and reliable therapeutic targets for glioma patients.

Tumour angiogenesis has been revealed to be involved in mediating tumour growth and metastasis.^102^, ^103^ The high level of microvessel density can be identified as an independent prognostic indictor for glioma patients.^104^, ^105^ Ma et al identified SNHG15 as a novel lncRNA involved in the growth of glioma microvascular endothelial cells.^106^ Their study demonstrated that SNHG15 was significantly increased in glioma‐mediated human cerebral microvascular endothelial cells (hCMECs), which was cultured in the glioma conditioned medium (GCM) to simulate the glioma microenvironment.^106^ Conversely, miR‐153 expression was obviously decreased in glioma‐induced hCMECs, compared with that in the hCMECs in the primary astrocyte cell conditioned medium (ACM).^106^ Interestingly, SNHG15 knockdown can induce the inhibition of VEGFA and Cdc42, and miR‐153 knockdown can significantly upregulate the expression of VEGFA and Cdc42, which were recognized to activate angiogenesis.^103^, ^106^, ^107^, ^108^

Then, a series of functional assays were performed to clarify the biological effects of both SNHG15 and miR‐153 on proliferation, migration and tube formation of glioma vascular endothelial cells. They showed that decreased SNHG15 expression can suppress proliferative and migratory capacity of glioma vascular endothelial cells.^106^ As for tube formation, the results showed that knockdown of SNHG15 can effectively reduce the relative tubule length and relative number of branches, suggesting the promotion effects of SNHG15 on tube formation of glioma vascular endothelial cells.^106^ As expected, glioma patients with high SNHG15 expression are tended to have poorer OS, while the glioma patients with high miR‐153 level are more likely to have better OS.^106^ However, more analysis, such as univariate analysis and multivariate Cox regression model, should be done to identify independent factors associated with glioma patients. Ma et al disclosed that lncRNA SNHG15 mediates glioma vascular endothelial cells through SNHG15/miR‐153/VEGFA/Cdc42 axis, presenting as therapeutic target for glioma therapy against angiogenesis.

Moreover, a recent study also revealed the role of lncRNA SNHG15 in the tumour microenvironment (TME). High level of SNHG15 was shown to be associated with a significantly higher risk of developing GBM, which represents the largest and most lethal subgroup of brain tumours.^109^ Li et al also found that knockdown of SNHG15 can obviously inhibit tumorigenesis, self‐renewal and elevate temozolomide (TMZ) sensitivity.^110^ It is determined that TMZ‐resistant (TMZ‐R) GBM cells are able to promote M2‐polarization of glioma associated microglia (GAMs), which are functionally similar to tumour‐associated macrophages in the peripheral system and interact with GBM cells through intracellular communications.^110^ Intriguingly, the treatment of palbociclib, CDK6 inhibitor, can effectively decrease the generation of M2 GAM and glioma stem cells mediated by TMZ‐R cells through downregulating SNHG15 and upregulating miR‐627.^110^ Consistently, M1 markers (IFN‐γ and TNF‐α) were prominently increased in the GAMs co‐cultured with SNHG15‐silenced TMZ‐R cells, whereas M2 markers (IL‐6 and TGF‐β) were significantly decreased in the GAMs co‐cultured with SNHG15‐silenced TMZ‐R cells.^110^ Overall, the molecular axis of SNHG15/CDK6/miR‐627 may help to overcome the resistance of TMZ, supporting the usage of palbociclib for TMZ‐resistant GBM cases.^110^

### 3.10 Breast cancer

Breast cancer (BC) is the most commonly diagnosed cancer and the leading cause of cancer death among females, accounting for almost 1 in 4 cancer cases among women.^110^ It is predicted to be about 2.1 million newly diagnosed female BC cases in 2018.^61^ It is quite hard to modify the primary risk factor of BC due to prolonged period of endogenous hormonal exposures.^61^, ^111^ The implication of comprehensive treatment enables some BC patients to have relatively good clinical outcome.^111^, ^112^, ^113^ However, approximately a third of breast cancer patients may have relapses, metastasis and chemotherapy resistance.^112^, ^113^ The current situation of BC highlights the importance of new effective biomarker early detection and treatment. Thus, there is an imperative need for the development of novel therapies based on the biological and molecular mechanisms of breast cancer.

Kong et al investigated the expression pattern of SNHG15 in 58 BC tissues and 19 adjacent normal tissues.^114^ It was found that SNNHG15 was overexpressed in breast cancer tissues. Ectopic SNHG15 expression was found to be remarkably associated with TNM stage, lymph node metastasis in BC patients.^114^ And there was no significant correlation between SNHG15 level and age of BC patients.^114^ Kaplan‐Meier analysis showed that breast patients with higher SNHG15 expression was positively correlated with poor survival.^114^ In‐vitro and in‐vivo assays both validated that SNHG15 knockdown can efficiently suppress cell proliferation, enhanced apoptosis and inhibit migration and invasion of BC cells.^114^ Moreover, their study revealed that SNHG15 knockdown can decrease vimentin expression and increase E‐Cadherin in BC cells.^114^ It suggested that the pro‐metastatic function of SNHG15 was determined to be correlated with EMT regulation. Mechanistically, Kong et al proved that SNHG15 can bind with miR‐211‐3p, which was determined to be significantly downregulated in BC tissues.^114^ And a negative correlation was determined between SNHG15 expression and miR‐211‐3p expression in BC tissues.^114^ Besides, rescue assays demonstrated that miR‐211‐3p downregulation can partly reversed the inhibition of tumorigenesis induced by SNHG15 knockdown in BC cells.^114^ Kong and his colleagues uncovered that lncRNA SNHG15 exerts oncogenic role through negatively regulating miR‐211‐3p in BC progression, which may give new insight into molecular diagnosis and treatment.^114^ However, the sample size in this study is limited and the listed clinical parameters only include tumour size, TNM stage, lymph node metastasis and age. In the future, more efforts should be made to determine the underlying mechanism and clinical value of SNHG15 in BC.

### 3.11 Renal cell carcinoma

Renal cell carcinoma (RCC), as the most common type of kidney cancer, accounts for 2%‐3% of all cancer cases worldwide.^115^ It is estimated to have approximately 403 262 new cases and 175 098 deaths in 2018.^61^ At present, even modern imaging modalities, such as computed tomography or magnetic resonance imaging, often failed to precisely distinguish between benign and malignant renal tumours, setting up unnecessary surgical operations.^116^ Unfortunately, there was still no effective biomarkers of kidney cancer patients for conventional screening, diagnosis, monitoring and therapeutics.^116^, ^117^ Therefore, prospective biomarkers are urgently needed to facilitate early detection and precise treatment by implementing underlying mechanism of kidney cancer.

Du et al identified SNHG15 as an overexpressed lncRNA in RCC tissues through analysing the publicly available data from TCGA and GEO data sets.^116^, ^117^ Then, the expression of SNHG15 was further validated in a cohort of 96 pairs RCC tissues and matched non‐cancerous tissue samples. It was found that lncRNA SNHG15 was obviously upregulated in RCC tissues, compared with control group.^18^ Elevated SNHG15 level was found to be positively correlated with histological differentiation and T stage, revealing that SNHG15 may be a candidate marker for RCC patients.^18^ Meanwhile, the prognostic information of RCC patients was obtained from the OncoLnc website, and RCC patients were divided into high SNHG15 expression group and low SNHG15 expression group according to the median value. The survival analysis showed that RCC patients with higher SNHG15 expression level are tended to have worse prognosis.^18^ Similarly, SNHG15 was obviously increased in RCC cells, compared with the normal renal cell line HK‐2.^18^ Functional investigation revealed that decreased SNHG15 expression can effectively suppress cell proliferation, migration and invasion, cause G1/G0 arrest and induce apoptosis in RCC.^18^ Moreover, SNHG15 knockdown can regulate EMT process, presenting as reduction of N‐cadherin and Vimentin and increase of E‐cadherin.^18^ Specifically, SNHG15 can regulate NF‐κB signalling pathway, thus contributing to renal cell carcinoma proliferation and EMT.^18^ This mechanistic model may shed new light on RCC pathogenesis and molecular treatment. However, the number of cases with lymphatic invasion or metastasis was too small to perform statistical analysis.

### 3.12 Ovarian cancer

Epithelial ovarian cancer (EOC) is the second most common gynaecological malignancy in women worldwide, accounting for a third of all gynaecological malignant tumours.^118^ Due to late clinical presentation and lack of sensitive and specific biomarkers, it is extremely hard to detect ovarian cancer at early, leading to a high rate of metastasis and recurrence.^118^, ^119^ Thus, identifications of effective biomarkers for diagnosis and therapeutics would be of great clinical significance for ovarian cancer patients.

Qu et al measured the expression level of SNHG15 in EOC tissues and cells and revealed the overexpression of SNHG15 in both EOC tissues and cell lines.^35^ Interestingly, patients with Type II cancers showed higher SNHG15 levels than patients with Type I cancers.^35^ Further analysis showed that high SNHG15 expression was closely relevant to cancer type, ascites and FIGO stage, revealing the clinical potential of SNHG15 in EOC.^35^ Based on the survival analysis, univariate analysis, and multivariate Cox regression model, SNHG15 was identified to be positively correlated with poorer overall survival and DFS, serving as an independent risk factor for poor OS and PFS in EOC patients.^35^ Functionally, knockdown of SNHG15 can notably suppressed migration and invasion of EOC cells.^35^ Meanwhile, downregulation of SNHG15 can inhibit the proliferative capacity of EOC cells, while ectopic expression of SNHG15 can exert promoting effects on EOC cell proliferation, migration and invasion.^35^ Besides, overexpression of SNHG15 can significantly elevate the inhibition rate of cisplatin, contributing to the chemoresistance of EOC cells.^35^ In summary, Qu et al discovered that SNHG15 acts as an oncogene and suggests its utilities as prognostic indicator of EOC patients. However, larger tumour samples and deep mechanistic research are urgently needed.

## 4 MECHANISTIC MODEL OF SNHG15 IN HUMAN CANCER

**Figure 1:**
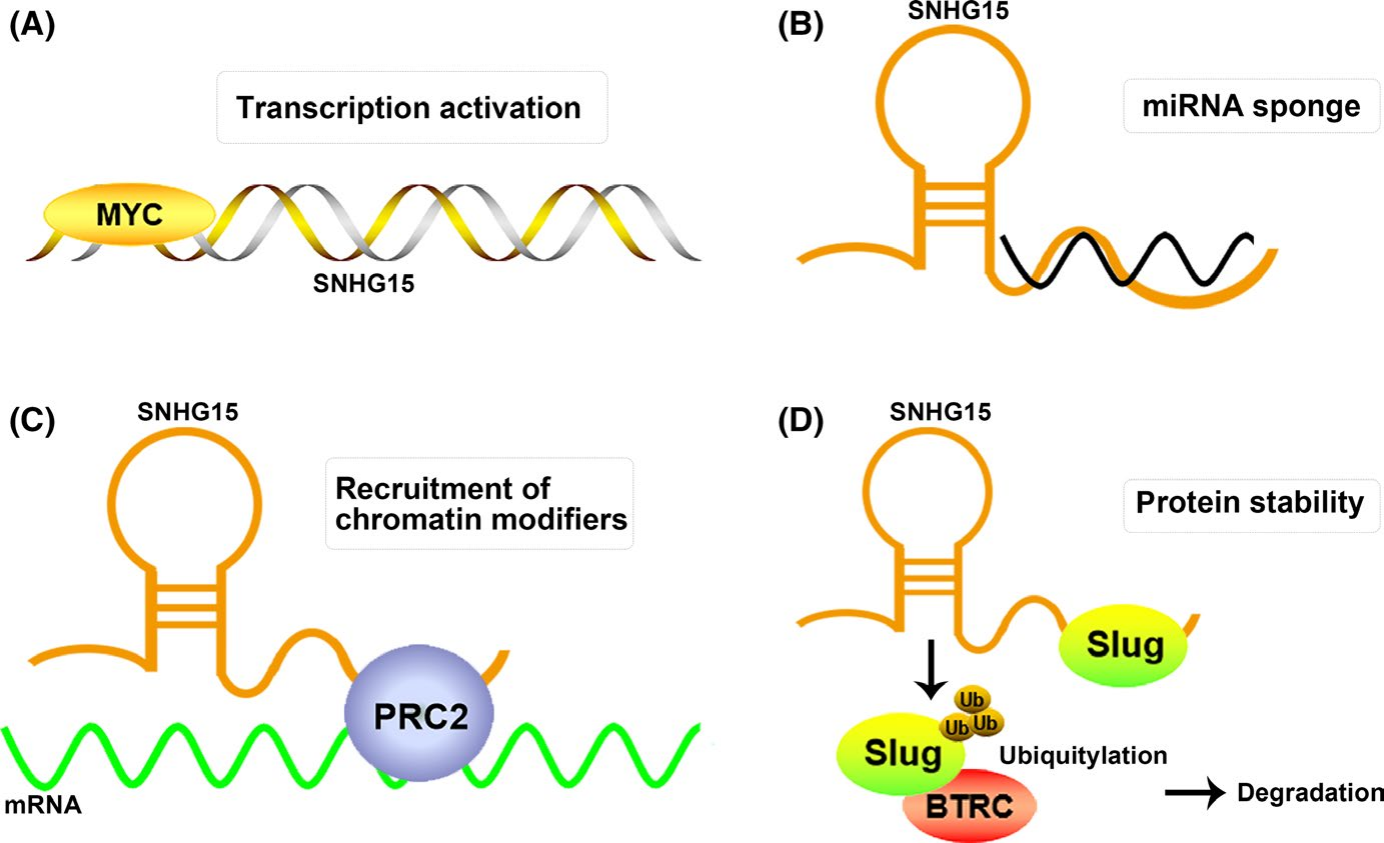
Mechanistic model of SNHG15 in human cancer. (A)The transcription of SNHG15 can be significantly activated by oncogenic MYC. (B) SNHG15 could function as a ceRNA to sponge miRNA in human cancers. (C) SNHG15 could recruit PRC2 to the promoter of key target, leading to gene silencing and cancer promotion. (D) SNHG15 could bind with Slug and influence its stability through UPP

Recent progress has indicated that lncRNA SNHG15 could exert oncogenic or tumour‐suppressive function in various cancers through various regulatory mechanisms (Figure 1). Better understanding of the mechanistic model of SNHG15 in human cancers may give new insight into cancer pathogenesis and lncRNA‐based therapeutics.

### 4.1 Upstream regulator essential for aberrant expression of SNHG15

Mounting evidences have demonstrated that transcription factors, and genetic alterations can lead to the aberrant expression of lncRNAs.^120^, ^121^ Previous study declared that there are two E‐box (CACGTG) binding motifs for transcription factor MYC on the first exon and first intron of SNHG15.^122^ Analysis of ChIP‐seq data from ENCODE confirmed that transcription factor MYC can bind to the mentioned E‐box in various cancerous cells.^49^ Saeinasab et al further explored the RNA‐seq data correlated with colorectal adenocarcinoma from TCGA data set and found that SNHG15 displayed significant upregulation in CRC tissues with highly expressed MYC expression.^49^ Consistently, knockdown of MYC can significantly decreased SNHG15 expression in CRC cells, illuminating that MYC can activate the transcription of SNHG15 in CRC cells.^49^ In summary, MYC is involved in the transcription of SNHG15 overexpression. However, more upstream modulators need to be uncovered in the future.

### 4.2 SNHG15 as ceRNA with potential roles in the context of post‐transcriptional regulation

**Figure 2:**
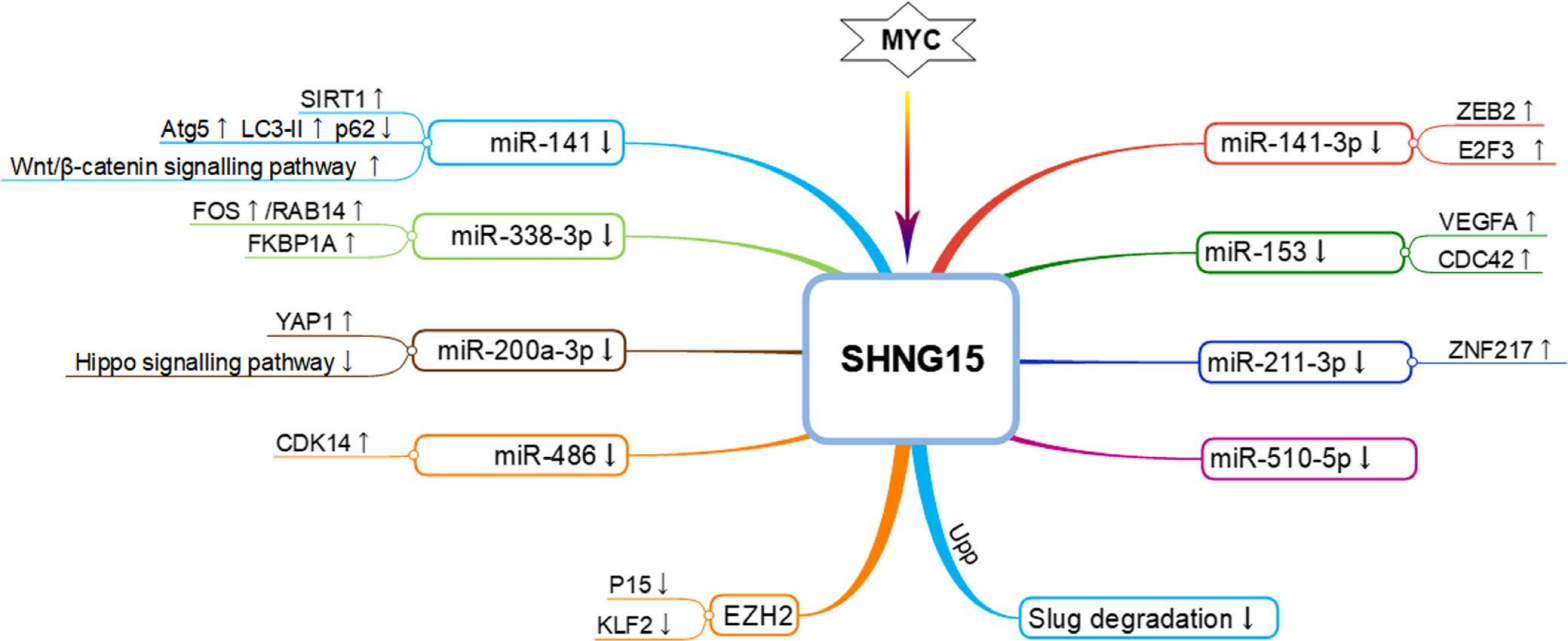
SNHG15‐involved ceRNA network in cancer progression

It is currently known that the transcripts, which harbour miRNA response elements (MREs), may have a potential to act as ceRNAs, including lncRNAs, pseudogenes, circular (circ)RNAs and mRNAs.^123^, ^124^, ^125^, ^126^, ^127^ The theory of ceRNA implies that there are regulatory networks lying foundation for crosstalk between ncRNAs and coding RNAs through miRNA involvement.^128^, ^129^ Particularly, lncRNA‐miRNA‐mRNA network has been shown to play critical roles in the development and progression of various neoplasms.^130^, ^131^ LncRNA SNHG15, as an essential regulator in human cancer, has been elucidated to be involved in ceRNA network, thus impacting biological and pathological activities in cancer progression (Figure 2).

#### 4.2.1 LncRNA SNHG15/miR‐338‐3p/mRNA FKBP1A

Zhang et al reported that SNHG15 can act as a ceRNA to regulate miRNA‐mRNA axis in prostate cancer.^30^ This study indicated the miR‐338‐3p silencing through SNHG15, which holds binding elements for miR‐338‐3p at its 3′‐UTR, leading to ectopic expression of FKBP1A.^30^ The overexpression of miR‐338‐3p can significantly decrease the luciferase activity of the wild‐type FKBP1A, but exhibit no influence on the mutant type FKBP1A.^30^ In summary, SNHG15 elevated FKBP1A expression by sponging miR‐338‐3p at post‐transcriptional level, thus regulating the biological processes of prostate cancer.

#### 4.2.2 LncRNA/miR‐338‐3p/mRNA FOS‐RAB14

Li et al elucidated that SNHG15 can stimulate CRC proliferation through miR‐338‐3p/FOS/RAB14 axis.^50^ Mechanistic study showed that SNHG15 could directly interact with miR‐338‐3p, which can directly target FOS‐RAB14 and exert anti‐tumour functions in CRC.^50^ Ectopic expression of SNHG15 can significantly restored the inhibitory effects on proliferation and promoting effects on apoptosis mediated by miR‐388‐3p.^50^ Moreover, there was a positive correlation between SNHG15 level and FOS‐RAB14 in CRC tissues.^50^ Of note, knockdown of FOS and RAB14 can impair the proliferation promotion mediated by SNHG15 overexpression or miR‐388‐3p silencing.^50^ These findings support the notion of SNHG15/miR‐388‐3p/FOS/RAB14 in CRC tumorigenesis.

#### 4.2.3 LncRNA SNHG15/ miR‐141/mRNA SIRT1

Another ceRNA study of SNHG15 in the CRC development via sponging miR‐141.^19^ In this study, SNHG15 was found to act as ceRNA for decreasing miR‐141, resulting in the overexpression of SIRT1.^19^ Moreover, further research validated that knockdown of SNHG15 can effectively suppressed the protein level of wnt1, c‐myc, cyclin‐D1 and β‐catenin, which are critical proteins correlated with wnt/β‐catenin signal.^19^ Consistently, decreased miR‐141 expression can obviously reverse the suppressive effects of wnt/β‐catenin signal‐related proteins induced by SNHG15 knockdown.^19^ Collectively, SNHG15 can play an oncogenic role in CRC development via brisking wnt/β‐catenin signal and the regulatory network mediated by SNHG15/miR‐141/SIRT1 axis can pave a new sight in understanding CRC biology.^19^ As for osteosarcoma, the mechanistic model of SNHG15/ miR‐141 was also identified by Liu et al^31^ SNHG15 was found to be directly interact with miR‐141 and negatively regulate mi4‐141 expression.^31^ Consistently, overexpression of miR‐141 can obviously attenuate tumorigenesis and autophagy directed by SNHG15 overexpression in OS cells.^31^ The SNHG15/miR‐141 axis may open a new window for understanding the hidden aspects of OS.

#### 4.2.4 LncRNA SNHG15/ miR‐486/mRNA CDK14

Jin et al found that SNHG15 induced NSCLC tumorigenesis and metastasis through activating CDK14 expression via competitively binding with miR‐486.^44^ The ceRNA role of SNHG15 is responsible for the progression of NSCLC cells through suppressing miR‐486, which can decrease CDK14 expression.^44^ These findings highlighted that SNHG15 as essential regulator of SNHG15/ miR‐486/ CDK14 axis plays key roles in NSCLC progression and metastasis.^44^

#### 4.2.5 LncRNA SNHG15/miR‐211‐3p/ mRNA ZNF217

Kong et al have clarified the oncogenic function SNHG15 in regulating cell proliferation, migration and EMT by acting as ceRNA to sponge miR‐211‐3p.^114^ In contrast, miR‐211‐3P was decreased in BC tissues and cells and exhibited tumour‐suppressive roles in BC.^114^ Notably, there was an opposite correlation between SNHG15 and miR‐211‐3p in BC tissues.^114^ Currently, a continuing research in NSCLC reported another ceRNA network linked to SNHG15‐miR‐211‐3p‐ZNF217.^43^ SNHG15 could serve as a ceRNA to upregulate ZNF217 expression, contributing to NSCLC tumorigenesis and metastasis.^43^ Furthermore, the inhibitory effect of miR‐211‐3p on NSCLC proliferative and migratory potential can be partly rescued by overexpression of ZNF217.^43^ Thus, SNHG15‐involved ceRNA network may be of great significance to identification of biomarker or therapeutic targets for patients with BC or NSCLC.

#### 4.2.6 LncRNA SNHG15/miR‐153/mRNA VEGFA/Cdc42

One of the key regulatory mechanism linked to glioma is the lncRNA SNHG15/ miR‐153/VEGFA/Cdc42 ceRNA network revealed by Ma et al^106^ Overexpression of SNHG15 play a promoting role in regulating glioma vascular endothelial cell proliferation, migration and tube formation through competitively sponging miR‐153.^106^ And miR‐153 can directly target the 3'‐untranslated region of VEGFA and Cdc42, leading to obvious downregulation of VEGFA and Cdc42.^106^ It was notable that miR‐153 enrichment can obviously reverse SNHG15‐mediated promoting effects of cell proliferation, migration and tube formation and knockdown of SNHG15 combined with overexpression of miR‐153 can efficiently inhibit angiogenesis of glioma vascular endothelial cells.^106^ Thus, SNHG15 may be involved in the biological behaviours of glioma vascular endothelial cells through SNHG15/ miR‐153/VEGFA/Cdc42 axis.

#### 4.2.7 LncRNA SNHG15/miR‐141‐3p/mRNA ZEB2/E2F3

Recently, Ye et al reported the specific function and molecular mechanism of SNHG15 in hepatocellular carcinoma.^74^ SNHG15 was shown to exhibit oncogenic properties in hepatocellular carcinoma (HCC) by sponging tumour‐suppressive miR‐141‐3p, in turn, increasing ZEB2 and E2F3 expression.^74^ Furthermore, the suppression of miR‐141‐3p can partially reverse the inhibitory influence of HCC carcinogenesis driven by SNHG15 knockdown.^74^ The interplay of SNHG15/miR‐141‐3p/mRNA ZEB2/E2F3 in HCC may add another layer of ceRNA regulation, facilitating the understanding of HCC pathogenesis.

#### 4.2.8 LncRNA SNHG15/miR‐200a‐3p/mRNA YAP1

Wu et al revealed that SNHG15 could act as a ceRNA by competitively binding with miR‐200a‐3p in PTC cells, thus upregulating the expression of YAP1, which was a key downstream regulator of Hippo signalling pathway.^33^ Wu and his colleagues have demonstrated that SNHG15 exerts pro‐oncogenic roles in regulating cell proliferation, migration and EMT progress in PTC.^33^ The distribution of SNHG15 is mainly located in the cytoplasm, which highlights the regulatory mechanism of SNHG15 at post‐transcriptional level.^33^ There was a negative association between SNHG15 and miR‐200a‐3p expression in PTC tissue samples.^33^ Further research revealed that overexpression of miR‐200a‐3p can significantly decrease the expression level of YAP1, which was also positively regulated by SNHG15 in PTC cells.^33^ Intriguingly, the suppressive effects of PTC cell proliferation, migration and EMT process directed by SNHG15 knockdown can be partly reversed by YAP1 overexpression or miR‐200a‐3p downregulation.^33^ In summary, the SNHG15/miR‐200a‐3p/YAP1 can mediate PTC progression, shedding new light on PTC diagnosis and molecular therapeutics for PTC patients.

### 4.3 SNHG15 and EZH2 interaction at transcriptional level

A rapidly growing body of data have indicated that lncRNA could interact with the Polycomb‐repressive complex 2, which can mediate histone methylation and mainly consists of EZH2, SUZ12 and EED.^132^, ^133^, ^134^ EZH2, as a critical component of PRC2, can catalyses H3K27me3 and closely correlates with tumorigenesis.^135^ The interplay between EZH2 and DNA methyltransferases (DNMTs) links H3K27 and CpG methylation leading to DNA hypermethylation and consequent silencing of genes.^135^ And lncRNAs can induce epigenetic activation or gene expression silencing via interacting with RNA‐binding proteins (RBPs).^134^, ^136^ Currently, Ma et al demonstrated that SNHG15 can bind to EZH2 and SUZ12 and play oncogenic roles in pancreatic cancer.^41^ Extensive studies have validated the expression pattern of EZH2 in various tumour tissues, including pancreatic cancer.^41^, ^137^, ^138^ EZH2 was significantly increased in PC tissues, compared with matched non‐cancerous tissue samples.^139^ Similar to the promotion effects of SNHG15 on PC cell proliferation, knockdown of EZH2 can efficiently inhibit the proliferative capacity of PC cells.^139^ Moreover, SNHG15 expression was much higher in nucleus compared with that in cytoplasm, suggesting the potential regulatory mechanism of SNHG15 at transcriptional level.^41^ Interestingly, the expression level of P15 and KLF2 displayed obvious upregulation in pancreatic cells with knockdown of SNHG15 or EZH2.^41^ The co‐regulatory effects of SNHG15 and EZH2 on the expression of P15 and KLF2 indicated that SNHG15 may modulate the expression of P15 and KLF2 through recruitment of EZH2. Further research revealed that SNHG15 downregulation can lead to decreased EZH2 binding and H3K27me3 occupancy of the P15 and KLF2.^41^ These findings indicated that lncRNA SNHG15 can repress P15 and KLF2 through EZH2‐mediated H3K27me3, thus contributing to PC cell proliferation.

### 4.4 SNHG15 involvement in controlling protein stability

Current evidences have revealed roles of several lncRNAs in the post‐transcriptional regulation of gene stability.^140^, ^141^ Jiang et al recently uncovered that SNHG15 maintains Slug stability in living cells by impeding its ubiquitination and degradation through interaction with the zinc finger domain of Slug.^51^ It was found that SNHG15 can activate the protein level of endogenous Slug, but exhibited no influence on expression level of Slug at mRNA level.^51^ The specific interaction between SNHG15 and Slug zinc finger domain in mammalian cells was confirmed by RNA‐pull down, mapping assays and RNA immunoprecipitation (RIP) assays.^51^ Interestingly, The Slug stability mediated by SNHG15 was determined to correlate with proteasome‐mediated degradation.^51^ Specifically, Slug protein level was upregulated in cells treated with overexpression of SNHG15 and proteasome inhibitor MG132, while the protein level of Slug was obviously downregulated in cells with absence of SNHG15 and MG132.^51^ Consistently, overexpression of SNHG15 can significantly increase the half‐life of Slug, while knockdown of SNHG15 display negative effects.^51^ As for Slug ubiquitination, it can be effectively inhibited by SNHG15 overexpression and significantly activated by SNHG15 knockdown. LncRNA SNHG15 can mediate the stability of Slug protein through blocking its ubiquitination and proteasomal degradation.^51^ Specifically, lncRNA SNHG15 blocks BTRC‐mediated Slug ubiquitination by inhibiting the interaction between BTRC and Slug, thereby preventing Slug proteasomal degradation. β‐transducin repeatcontaining (BTRC). These findings revealed a novel mechanism underlying the control of Slug stability by demonstrating that oncogenic lncRNA SNHG15 interacts with and blocks Slug degradation via the ubiquitin‐proteasome system. Thus, SNHG15 is expected to serve as a target for CRC therapy.

## 5 THE INVOLVEMENT OF SNHG15 IN MULTIPLE SIGNALLING PATHWAYS

Recent discoveries have highlighted the importance of lncRNA SNHG15 in mediating various signalling pathways. The involvement of SNHG15 in multiple pathways in various cancers is listed in Table 2.

**Table 2:** The involvement of SNHG15 in multiple signalling pathways

Cancer type	Cell lines	Expression	Role	Related genes	Signalling pathways
Colorectal cancer	CaCO‐2, HCT‐116	Upregulated	Oncogenic	Wnt1, C‐Myc, Cyclin‐D1, β‐catenin, E‐cadherin, N‐cadherin, Vimentin, Snail	WNT/β‐catenin pathway
Renal cell carcinoma	ACHN, 786‐O	Upregulated	Oncogenic	NF‐kb, Snail1, Slug, ZEB1, N‐cadherin, Vimentin, E‐cadherin	NF‐kb signalling pathway
Gastric cancer	MGC803, BGC823	Upregulated	Oncogenic	MMP2, MMP9	EMT regulation
Lung cancer	A549	Upregulated	Oncogenic	MMP2, MMP9, E‐cadherin, N‐cadherin, Vimentin	EMT regulation
Colorectal cancer	HCT116, SW480,SW1116	Upregulated	Oncogenic	Slug, E‐cadherin	EMT regulation
Thyroid cancer	BCPAP, K1	Upregulated	Oncogenic	YAP1, MST1, LATS1	YAP‐Hippo pathway

### 5.1 Regulations of EMT signalling pathways in human cancers

Tumour metastasis is considered to be a major cause of dismal prognosis in patients suffered with cancer, in which many factors are involved. The initial step of this cascade is orchestrated by the induction of the EMT process, which is tightly regulated by multiple signalling pathways, such as WNT/β‐catenin, NF‐kB and TGF‐β signalling pathways.^55^, ^142^, ^143^, ^144^ Currently, lncRNAs have emerged in the regulation of EMT‐associated signalling pathways.

#### 5.1.1 WNT/β‐catenin signalling pathway in EMT

The WNT/β‐catenin signalling pathway has been recognized to be indispensable for EMT regulation, and its briskness is recurring encountered during the initiation and progression of various cancers, including CRC.^143^, ^144^, ^145^ A recent study conducted by Sun et al have confirmed the oncogenic regulatory axis of SNHG15/miR‐141/SIRT1/Wnt/β‐catenin pathway in CRC development.^19^ Sun and his colleagues pointed out that decreased SNHG15 expression can effectively inhibit the briskness of WNT/β‐catenin signalling pathway and EMT process in CRC development, with aberrant expression of EMT markers and WNT/β‐catenin pathway associated with proteins (Wnt1, C‐Myc, Cyclin‐D1 and β‐catenin).^19^ And these effects mediated by SNHG15 knockdown can be changeover by co‐transfection of miR‐141 inhibitor in CRC cells.^19^ However, further studies are needed to verify the regulatory relationship between SNHG15 and WNT/β‐catenin signalling pathway in CRC initiation and progression.

#### 5.1.2 NF‐kb signalling pathway in EMT

Accumulating studies have highlighted the critical role of NF‐kb signalling pathway in mediating EMT process, leading to cancer invasion and metastasis.^142^, ^146^ Du et al, reported that lncRNA SNHG15 contributes to EMT in RCC through regulating NF‐kb signalling pathway.^18^ It can be observed that SNHG15 knockdown can significantly decrease the nuclear fluorescence intensity of NF‐kb in RCC cells, with obvious decreased N‐cadherin and Vimentin expression and increased E‐cadherin expression.^18^ And the activation of NF‐kb signalling pathway can induce the expression of EMT‐associated markers, such as Snail1, Slug and ZEB1, which are also positively regulated by SNHG15 in RCC cells.^18^, ^147^ Therefore, this finding reported by Du et al revealed a plausible mechanism responsible for constitutive activation of NF‐κB signalling in migration and invasion of RCC, supporting the notion that SNHG15 may be a novel target for clinical treatment for RCC patients.^18^

#### 5.1.3 The common inducible factors in EMT

The process of EMT involves the decrease of epithelial marker (E‐cadherin, Zo‐1, and claudin1) and increase of mesenchymal marker (vimentin, ZEB1, N‐cadherin, Slug, Snail and NF‐κB).^148^ Moreover, it is known that the activation of NF‐κB can transcriptionally regulate the expression of MMP‐9, MMP‐2, uPA and VEGF, contributing to the acquisition of EMT phenotype.^149^ Current investigations have demonstrated that lncRNA SNHG15 can enhance malignant phenotypes through MMP2/MMP9 in both GC and NSCLC cells.^36^, ^40^ Interestingly, Jiang et al uncovered that lncRNA SNHG15 can interact with transcription factor Slug and keep its stability in living cells, thus regulating EMT and promoting colon cancer progression.^51^ Overall, it is notable that the existence of a novel mechanism by which lncRNA SNHG15 is integrated with the EMT‐associated signalling pathways to mediate the progression of multiple cancers, offering a novel rationale for lncRNA‐directed cancer therapeutics. However, the role and molecular basis of SNHG15 in other EMT‐associated signalling pathways still needs to be clarified in the future research.

### 5.2 YAP‐Hippo pathway

The Hippo signalling pathway is an evolutionarily conserved pathway, with YAP (Yes‐associated protein) as its main effector molecule.^150^ The inactivation of Hippo signalling pathway can lead to downregulation of MST1/LATS1 (the core factors of Hippo pathway) and upregulation of YAP1.^20^ The dysregulation of Hippo signalling has been recognized in a multitude of human tumours and closely associates with the acquisition of malignant traits.^20^ A recent study provided by Wu et al demonstrated that the regulatory axis of SNHG15/miR‐200a‐3p/YAP1 exerts oncogenic properties in PTC progression.^20^ Intriguingly, high level of SNHG15 can inactivate Hippo signalling pathway, with negatively correlated expression of MST1/LATS1 in PTC tissue samples.^33^ The current evidence for the existence of SNHG15/miR‐200a‐3p/YAP1/Hippo axis indicates that combined targeting of YAP1/Hippo signalling pathway may provide a potential direction for PTC treatment.^33^ However, extensive questions remain to be answered before transferring these notions into the clinical setting.

## 6 CONCLUSIONS AND FUTURE PERSPECTIVES

Extensive research has highlighted the critical roles of lncRNAs in tumour occurrence and progression.^151^, ^152^ The lncRNA SNHG15 has been revealed to be dysregulated in various cancers, and the expression trend of SNHG15 in various cancers are not completely consistent. SNHG15 is reported to significantly upregulated in most types of cancers and serves as an oncogenic regulator in cancer development and progression, including CRC, GC, HCC and PC.^40^, ^41^, ^42^, ^43^, ^74^ However, SNHG15 can also act as a tumour suppressor in TC tissues compared with normal tissues.^32^ The diversity of these studies can be partly explained by tumour heterogeneity, different gene expression background, different sources of tumour samples, as well as limited numbers of specimens. Moreover, aberrant expression of SNHG15 was determined to significantly related to some of clinical parameters and poor prognosis in may cancer patients, revealing its potential as an effective biomarker for cancer diagnosis and treatment.^34^, ^36^, ^42^ As a dysregulated lncRNA in multiple cancers, SNHG15 can impact various cellular activities, such as proliferation, migration, invasion, apoptosis, autophagy and metastasis.^34^, ^36^, ^42^ The molecular mechanism of SNHG15 revealed that the transcription of SNHG15 can be obviously activated by oncogenic transcription factors, leading to high expression of SNHG15 in malignant disease.^49^ SNHG15 could also function as a ceRNA which is sponging many different miRNA in cancer and consequently leading to the modulation of oncogenic factors, thus regulating malignant phenotype and mediating EMT.^18^, ^30^ Another mechanistic model of SNHG15 is shown to propose a general model for the EZH2 recruitment to chromatin through direct and specific interactions with SNHG15.^41^ Moreover, SNHG15 can also participate in regulating protein stability, thus controlling various biological processes.^51^ Current evidences have shown that SNHG15 is involved in some signalling pathways essential for cancer, including EMT, WNT/β‐catenin signalling, NF‐kb signalling pathways and YAP‐Hippo signalling pathways.^18^, ^19^ These signalling pathway involved in SNHG15‐mediated carcinogenesis may provide new strategy for tumour therapy.

In conclusion, SNHG15 can serve as an independent prognostic indicator in many cancers and may be a prospective and effective biomarker for cancer diagnosis and treatment. Despite mounting studies to illuminate the biological function and molecular mechanism of SNHG15 in various cancers, the current work is still at preliminary stage. Importantly, the lack of rescue assay is a major pitfall for existing published papers of SNHG15. In the future, more tissue samples should be used to further determine the expression pattern of SNHG15 in different cancers and further clarify the correlation among SNHG15 level, clinicopathological characteristics and prognosis of cancer patients. Multiple effects between SNNHG15 and molecular targets should be explored in depth, thus facilitating the clinical implication. Furthermore, the expression pattern and molecular mechanism of SNHG15 in body fluids are completely unknown, which are also needed.

## CONFLICT OF INTEREST

The authors declare that they have no competing interests.

## AUTHORS' CONTRIBUTIONS

You Shuai and Zhonghua Ma collected and summarized current evidences and progress. Jianwei Lu and Jifeng Feng guided the overall topic and writing. 

## Data Availability

Research data are not shared.
